# Our Buffalo Resolutions on Tuberculosis

**Published:** 1896-12

**Authors:** 


### OUR BUFFALO RESOLUTIONS ON TUBERCULOSIS.

EVERY veterinary organization in the land would do well to take up at the earliest opportunity the resolutions adopted by the United States Veterinary Medical Association at Buffalo, and, after thorough consideration, place themselves on record in their communities. These resolutions go right to the root of the whole subject, and are conservative in every direction where conservatism is justified. They are broad in the highest aspect of the subject from a veterinary and sanitary point of view, and they should be given the utmost publicity throughout the land. They were adopted unanimously at Buffalo by the largest and most representative convention of veterinarians ever assembled on this continent, embracing delegates from the United States and Canada, and these will do more to advance the movement for wiser laws for the control of *this disease*, when studied by an enlightened public, than any expression of views on this subject uttered during the past ten years. Advance the movement!

---

"BACTERIOLOGY in its Relation to Veterinary Science," will be the subject of an address by Dr. M. P. Ravenal, Bacteriologist of the Pennsylvania State Veterinary Sanitary Board, at the meeting of the Keystone Veterinary Medical Association on December 8, 1896. An invitation is extended to all veterinarians to be present.

